# Subcellular functions of tau mediates repair response and synaptic homeostasis in injury

**DOI:** 10.21203/rs.3.rs-3897741/v1

**Published:** 2024-02-29

**Authors:** David Grosshans, Riya Thomas, Die Zhang, Christopher Cronkite, Rintu Thomas, Sanjay Singh, Lawrence Bronk, Rodrigo Morales, Joseph Duman

**Affiliations:** MD Anderson Cancer Center; The University of Texas Health Science Center at Houston

## Abstract

Injury responses in terminally differentiated cells such as neurons is tightly regulated by pathways aiding homeostatic maintenance. Cancer patients subjected to neuronal injury in brain radiation experience cognitive declines similar to those seen in primary neurodegenerative diseases. Numerous studies have investigated the effect of radiation in proliferating cells of the brain, yet the impact in differentiated, post-mitotic neurons, especially the structural and functional alterations remain largely elusive. We identified that microtubule-associated tau is a critical player in neuronal injury response via compartmentalized functions in both repair-centric and synaptic regulatory pathways. Ionizing radiation-induced injury acutely induces increase in phosphorylated tau in the nucleus and directly interacts with histone 2AX (H2AX), a DNA damage repair (DDR) marker. Loss of tau significantly reduced H2AX after irradiation, indicating that tau may play an important role in neuronal DDR response. We also observed that loss of tau increases eukaryotic elongation factor levels after irradiation, the latter being a positive regulator of protein translation. This cascades into a significant increase in synaptic proteins, resulting in disrupted homeostasis. Consequently, novel object recognition test showed decrease in learning and memory in tau-knockout mice after irradiation, and electroencephalographic activity showed increase in delta and theta band oscillations, often seen in dementia patients. Our findings demonstrate tau’s previously undefined, multifunctional role in acute responses to injury, ranging from DDR response in the nucleus to synaptic function within a neuron. Such knowledge is vital to develop therapeutic strategies targeting neuronal injury in cognitive decline for at risk and vulnerable populations.

## Introduction

Brain tumors are the second leading cause of death in children and are a significant health challenge worldwide [1–5]. Depending on tumor type, size, location and patient age, radiation therapy is often used to improve local control and survival [6–11]. However, brain radiation can lead to cognitive declines similar to those observed in neurodegenerative patients [12, 13]. There is a growing urgency to elucidate the mechanisms behind this long-term sequelae of radiation treatment. Previous studies have focused on the effects of radiation injury in replicative neuronal precursor cells [14, 15], but terminally differentiated neurons are also impacted by undergoing structural and subsequent functional alterations [16, 17].

Owing to their post-mitotic and radiation-resistant characteristics, neurons were commonly viewed as passive bystanders in radiation-induced damage [18, 19]. However, there has been an increase in emphasis on understanding the integral role of the neuron in generating orchestrated responses for optimal brain function in the presence of an insult or injury [20]. Evolutionarily, neuronal architecture in the form of axon extensions, dendritic branches, and spine modifications exhibit robust repair with localized translation processes, sustaining survival and network communications across brain regions [21–23]. The neuronal cytoskeleton is a critical component of this machinery and microtubule-associated proteins are functionally critical for transporting mitochondria across axons [24, 25] and aiding in synaptic communication [26–28]. Among these proteins, microtubule-associated tau has emerged as a crucial player in tubulin assembly and cytoskeletal stabilization [29–31], and recent reports describe it as having both nuclear [32–35] and synaptic functions [36–38].

Studies have shown tau’s involvement in DNA damage, and also hyperthermic stress across multiple cellular compartments [33, 34, 39–42]. However, the mechanisms regulating these differential tau functions remains elusive. Therefore, understanding how tau mediates neuronal response to injuries such as radiation will elucidate its role in homeostasis, and help identify novel pathways that may be targeted to avert radiation-induced damage. Our study revealed altered tau dynamics in neurons following radiation with decreased cytoplasmic tau and increased phosphorylated tau (pTau) in the nucleus. Through immunoprecipitation mass spectrometry (IP-MS) and chromatin immunoprecipitation sequencing (ChIP-Seq) on human induced pluripotent stem cell (hiPSC)-derived neurons, we elucidated tau-associated interactome and its genome-wide binding sites in response to radiation-induced damage. The findings highlight tau’s neuroprotective role and emphasize the substantial disruption of crucial homeostatic functions upon loss of tau expression, encompassing DNA damage repair (DDR) response and protein translation. Additionally, these observations were supported by demonstrating tau’s critical involvement in synaptic dynamics, neuronal ring rate, wave functions, and cognitive performance *in vivo*. In this study, we present tau’s previously undefined roles that emerge in response to neuronal injury.

## Materials and Methods

### Primary neuronal cultures

Cortical and hippocampal neurons were prepared from 15-to-17-day-old rat embryos grown in culture. After surgical dissection of 8–10 pooled embryonic cortex and hippocampus, cultures were mechanically digested via trituration and maintained at 37°C for up to 28 days for neuronal differentiation.

### Cerebral organoid preparation

hiPSCs were cultured following a 2D feeder independent protocol. After two passages, a single cell suspension of hiPSCs was used to plate 9000 cells/well in ultra-low-binding 96-well plates, which were allowed to coalesce into embryoid bodies for 6 days before being transferred to plates containing neural induction media. On day 16, the embryoid bodies were transferred to Matrigel droplets in cold parafilm to gel at 37°C, and subsequently transferred to neural differentiation media. The embryoid bodies were further agitated in an orbital shaker at 37°C to develop into cerebral organoids.

### Tau knockdown in hiPSC-derived cerebral organoids

A MAPT-specific SMART vector-inducible lentiviral shRNA containing an EF1a promoter, developed by Horizon Discovery, was transduced into hiPSC-derived cerebral organoids. After determining the functional viral titer in cells, shRNA induction was initiated at doxycycline doses ranging from 0.1 μg/mL to 1.0 μg/mL. Induction of the Tet-On 3G system used in the SMART vector allowed the MAPT gene to be silenced upon doxycycline induction. Effective MAPT knockdown was further evaluated by cryosectioning frozen cerebral organoids on glass slides and immunoassayed for tau-specific antibodies.

### IP-MS

hiPSC-derived neural progenitors were differentiated to neurons and subjected to radiation dose of 10-Gy (treatment condition) with unirradiated (0 Gy) as the control. Proteins from neurons were extracted at 2 and 24 hours following irradiation and immunoprecipitated (IP) with ptau Ser_202_, Thr_205_ antibody, (#ab210703, Abcam). IP samples were trypsinized and digested for mass spectrometry analysis. Peptide spectrum matches were analyzed by Amanda Software and categorized by highest to lowest detections compared to unirradiated control. Gene Ontology and Metascape were used for proteomic analysis.

### ChIP-Seq

hiPSC-derived neurons were prepared and irradiated as for the IP-MS assay. Cells were fixed with formaldehyde solution and quenched with glycine. Samples were washed with PBS-Igepal, centrifuged, and pelleted. The snap-frozen pellets were processed for generating ChIPs with ptau Ser_202_,Thr_205_ antibody and subjected to Illumina sequencing (75-nucleotide single-end reads), library preparation, and analysis. Peaks were called by local enrichment within genomic regions in tag numbers via Model-based analysis of ChIP-Seq (MACS/MACS2). Sequencing reads were analyzed with Metascape to generate cluster maps.

### Animal care and preparation

In accordance with the guidelines of National Institutes of Health, all animals were treated per the Care and Use of Laboratory Animals Guide. MD Anderson Cancer Center’s Institutional Animal Care and Use Committee approved the study protocol. Male and female B6.129X1-*Mapt*^tm1Hnd^/J mice with knocked out microtubule-associated tau (Jackson Laboratory) and C57BL6 wild-type mice (as control) were used for all animal experiments. Mice were subjected to brain irradiation with a single 10-Gy dose and sham-irradiated mice were briefly anesthetized to serve as the unirradiated control. In accordance with animal experimental guidelines, brains were collected from mice 24 and 48 hours following irradiation. Dissected brain hemispheres were separated into cortex, hippocampus, and cerebellum before flash-freezing in liquid nitrogen for biochemical assays. The other brain hemisphere was processed for cryosectioning and immunofluorescence staining.

### Immuno uorescence (IF) staining and analysis

Neuronal cells, hiPSC-derived organoids, and mouse brain slices mounted on glass slides, were fixed with 4% paraformaldehyde. Samples were blocked for 1 hour with 2.5% BSA before incubating with primary antibodies at 4°C overnight. Primary antibodies used for IF were phospho-tau-AT8 (#MN1020, ThermoFisher), anti-tau-1 (#MAB3420, Millipore Sigma), eEF2, eEF2K, β-tubulin (#2332, #3692S, #2146S, Cell Signaling), Nestin (#NB100–1604, Novus). Samples were washed with PBS containing 0.1% TritonX-100 (PBST) prior to incubation with AlexaFluor secondary antibodies for 1 hour at room temperature. Images were captured on Cytation 5.0 under identical capture settings. High resolution images (60X silicon) were captured using the Andor Spinning-Disk Confocal microscope. Image analyses were conducted using QuPath open source software.

### Western blotting

Protein samples were separated on 4–12% Tris-glycine gels (Bio-Rad), transferred into PVDF membranes and blocked with 5% milk in TBS containing 0.1% Tween 20 (TBST). Membranes were incubated with primary antibodies at 4°C overnight. Antibodies used included phospho-tau-AT8, anti-tau-1, anti-puromycin (#MABE343, Millipore Sigma), phospho-Histone H2A.X (#9718, Cell Signaling), HISTSH2AC (#PA5–43691, Invitrogen), anti-PSD-95 (#1080-PSD95), anti-NMDA NR2A subunit (#1495-NR2A), anti-NMDA NR2B subunit (#1496-NR2B, phosphosolutions), BDNF (#47808S, Cell Signaling), GAPDH (#2118, Cell Signaling). Membranes were washed in TBST before probed with secondary antibodies for 1 hour at room temperature, then incubated with Femto ECL substrate and imaged on Bio-Rad Imaging System. Images were quantified using Fiji ImageJ.

### Synaptosome isolation

Mouse brain samples were homogenized in Syn-PER reagent (Cat#87793) and serially centrifuged at 1200xg for 10 min and 15,000xg for 20 min at 4°C to generate functional synaptosomes.

### Inhibitors

Ceralasertib (ADZ6738)-Ataxia-telangiectasia-mutated (ATR) inhibitor (Selleck Chemicals, Houston, TX, Cat#S7693) was dissolved at 5 mM stock.

### Surface tensing of translation (SUnSET)

SUnSET assay was conducted in *ex vivo* mouse brains maintained in fresh neuronal culture medium supplemented with puromycin (1 μM), modified from a protocol described in Goodman *et al* [43]. 30 min after incubation, brain tissue was processed for western blotting.

### Membrane cycling assay

Fluorescent styryl dye, N-(3-triethylammoniumpropyl)-(4-diethylamino)styryl)pyridinium dibromide, or FM^™^ 2–10 dye was used to test vesicle intake and exocytosis/endocytosis upon KCl activation. Luminescence measurements were recorded on spectrophotometer.

### Novel object recognition test

On day 1, mice were habituated in a white box for 20 min. On day 2, mice were placed into a white box containing two familiar objects and left to explore freely for 3 minutes. One hour later, mice were introduced back into the box with one of the familiar objects replaced with a novel object. Total exploration time (measured in seconds) for both familiar and novel objects was recorded for analysis.

#### In vivo electrophysiology recording

Mice were anesthetized with a combination of ketamine and xylazine combination and placed in a stereotaxic frame with body temperature maintained at 37°C by a homeothermic warming blanket. Extracellular recordings were obtained *in vivo* as described in our previous publications. In brief, glass electrodes (2M NaCl) were positioned in the prefrontal cortex (PFC) through a small burr hole in the skull by using coordinates (1.7 mm anterior to bregma, 0.5 mm lateral to the midline, 1.5–2.5 mm deep). A 5-min segment of spontaneous ring activity was recorded from each neuron. Fast Fourier transforms were used for these 5-min segments of local field potential waveforms at frequencies from 0.1 to 10 Hz to check the spectrum power distribution. Data were acquired with a bio-signal Zeus system and analyzed with Neuroexplorer. Electrodes were marked with Dil dye on their surfaces to identify the tracks left by extracellular microelectrodes. Data are reported only for those mice in which the electrodes were confirmed to be within the PFC regions.

### Statistical analysis

Data were analyzed by one-way or two-way analysis of variance followed by Tukey’s post hoc comparison or Student’s *t* test on GraphPad Prism; results are presented as mean +/− SEM. Statistical tests, number of samples, and p values are indicated on the figures.

## Results

### Neuronal injury induces nuclear sequestration of phosphorylated tau

**Figure 1:**
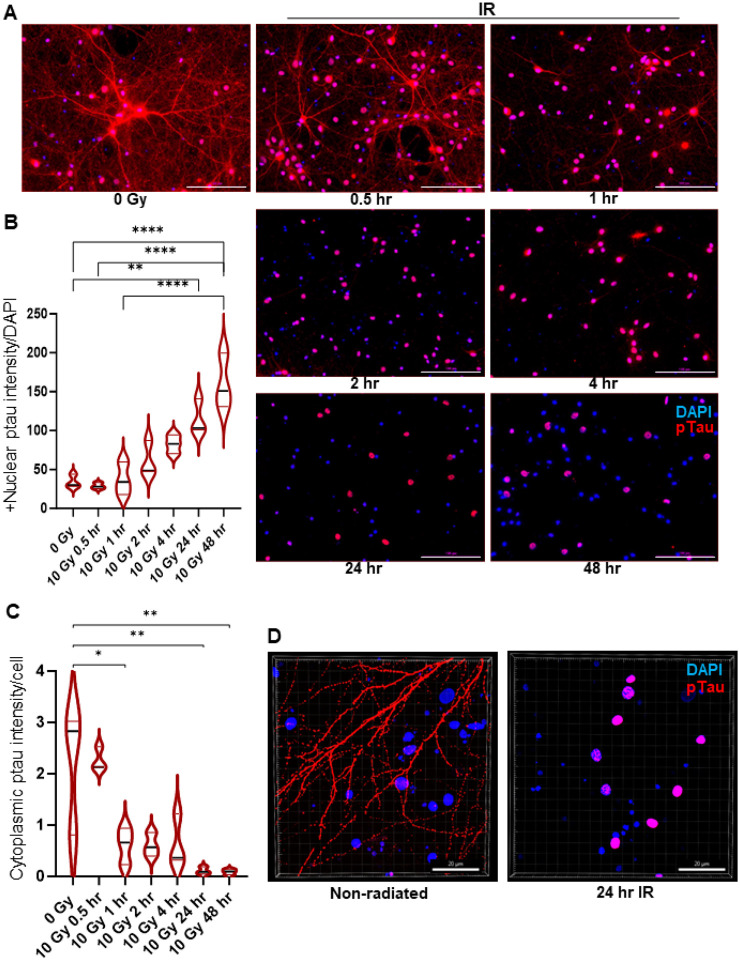
Neuronal injury induces nuclear sequestration of phosphorylated tau in primary cortical neurons. **A**ptau in irradiated versus unirradiated (0 Gy) control. Scale bar:100 μm. Quantitative analysis of nuclear (**B**) and cytoplasmic signal (**C**).**D** Imaris rendition of high resolution 60X images of non-radiated and irradiated neurons. n = 3. Data are represented as mean +/− SEM. 1-way analysis of variance. ****p<0.0001, **p<0.0021, *p<0.032.

Tau is phosphorylated at more than 85 different amino acid residues [44], which facilitates its dynamic binding capacity with microtubules [37]. We used IF to investigate ptau status after irradiation specifically at Ser_202_ and Thr_205_ residues in tau’s proline-rich middle domain, sites well characterized and established in mice and human brains with distinct tau-derived abnormalities [44]. Increase in nuclear ptau levels were observed as early as 0.5 hour after 10 Gy irradiation (compared to the unirradiated control), which persisted for up to 48 hours in primary rat cortical (Fig. 1A–B) neurons. In contrast, cytoplasmic ptau levels showed corresponding decrease in the neuronal cytoplasm as time progressed following irradiation (Fig. 1A,C). High resolution images further confirms this differential compartmentalization of ptau in irradiated cortical neurons (Fig. 1D). Similar patterns were also observed in hiPSC-derived cerebral organoids (Supplementary Fig. 1A-C). The increase in nuclear localization of ptau following radiation indicate tau’s potential role in radiation-induced DDR in neurons.

### ChIP-Seq and IP-MS identi es unique ptau-chromatin sites and ptau-interaction partners following radiation-induced neuronal injury

**Figure 2:**
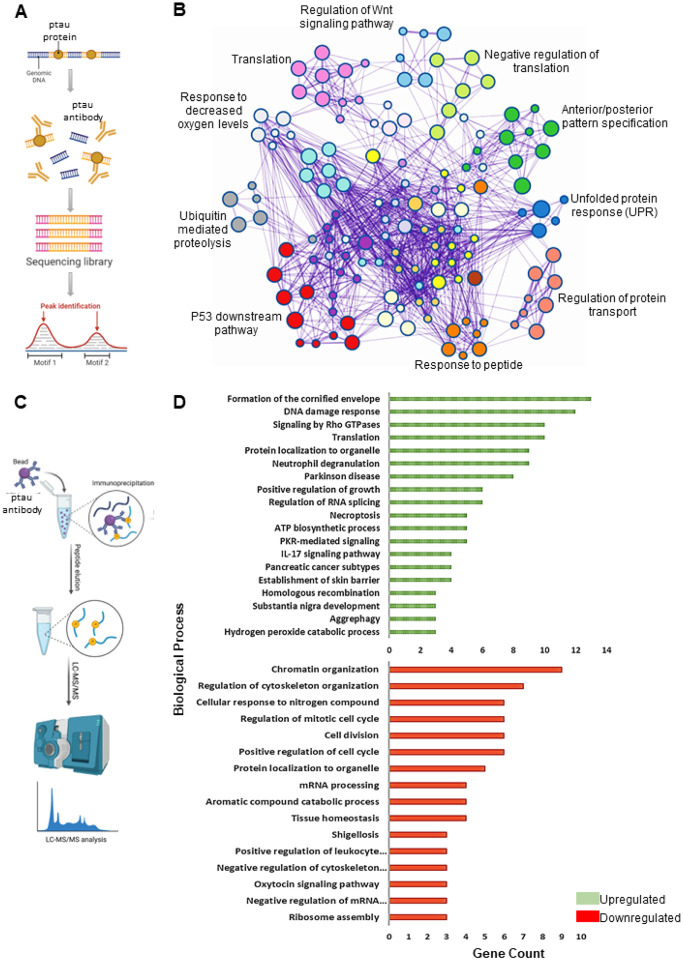
IP-MS and ChIP-Seq identifies unique ptau-interactors and ptau-chromatin sites following radiation-induced neuronal injury. **A** Experimental design of ChIP-Seq. **B** Metascape cluster analysis of differential gene expression across all timepoints after irradiation relative to control. **C**Experimental design of IP-MS. **D** Metascape count analysis of ptau-bound peptides classified into upregulated/downregulated categories after irradiation (relative to control).

To further investigate the direct binding of ptau to neuronal chromatin, ChIP-Seq was conducted on hiPSC-derived neurons (Fig. 2A). The results showed specific sites of direct ptau binding in the neuronal chromatin at 2 and 24 hours after irradiation. Subsequent analysis revealed ptau binding enrichment in genes regulating p53 regulated pathways critical for DDR, translation, protein transport, response to decreased oxygen levels, and negative regulators of translation (Fig. 2B). Peak locations relative to genomic annotations showed an increase in ptau enrichment in proximal promoters (0–1 kb), 5’-UTR, and exon regions (Supplementary Fig. 2A) at 2 and 24 hours after irradiation. These results revealed specific genomic regions where tau interacts with DNA, suggesting its potential involvement in DDR and other regulatory mechanisms like protein translation.

To assess changes in tau’s protein interactions following radiation, we conducted IP-MS of ptau in hiPSC-derived neurons (Fig. 2C). Peptides bound to ptau were categorized by biological processes into upregulated/downregulated across timepoints after irradiation relative to control. Upregulated categories included the cornified envelope, DNA damage response, signaling by Rho GTPases, and translation while downregulated categories involved chromatin organization, regulation of cytoskeletal organization, cellular response to nitrogen compound, and regulation of mitotic cell cycle (Fig. 2D). Gene ontology ranked DDR and translation as consistently enriched biological networks for ptau bound peptides at 2-hour and 24-hour timepoints following radiation. (Supplementary Fig. 3A). Peptide spectrum matching identified upregulated proteins like histone 2Ax (H2AX) and eukaryotic elongation factor 1A (eEF1A2), and downregulated proteins like eukaryotic elongation factor 2 (eEF2) at 2 hours post-radiation. Hence, the IP-MS data suggests that ptau exhibits altered protein interactions in response to radiation-induced neuronal injury. Notably, the identified proteins, including H2AX and eukaryotic elongation factors, point towards ptau’s role in DDR and translation mechanisms. These findings collectively emphasize the intricate involvement of ptau in orchestrating cellular responses to radiation-induced damage in neurons.

### DDR is diminished in brains of tau-knockout mice

**Figure 3:**
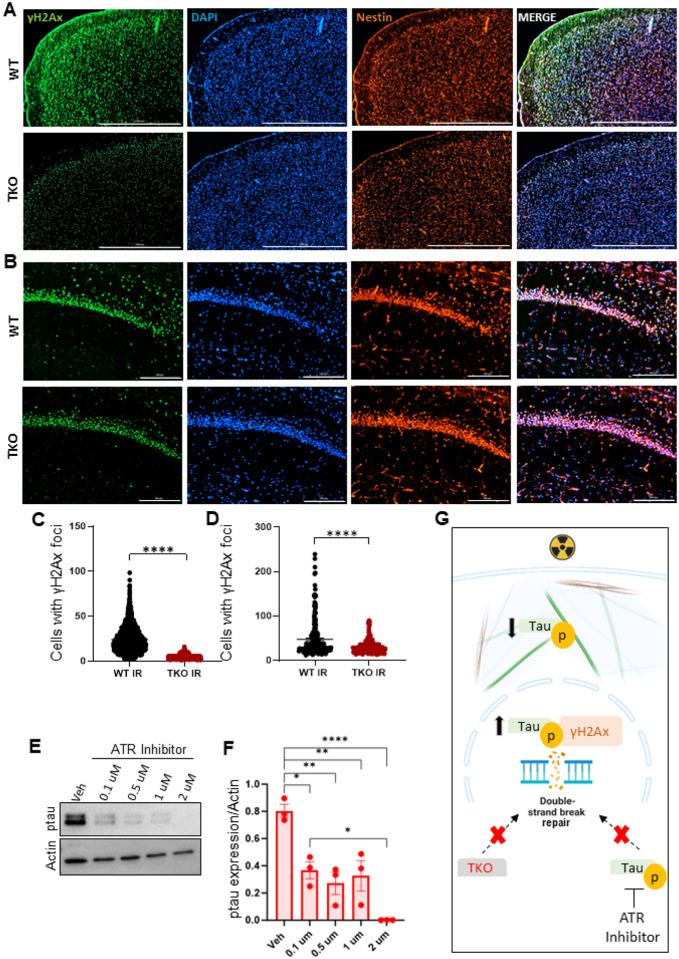
DNA damage repair is diminished in brains of tau-knockout mice. γH2Ax foci in the cortex (**A**) and CA1 hippocampus (**B**) of wild-type and tau-knockout (TKO) mice after irradiation. Scale bar:1000 μm (**A**). Scale bar:200 μm (**B**). Quantitative analysis of γH2Ax foci in cortex (**C**)and CA1 region of hippocampus (**D**). **E-F** Western blotting and analysis of neurons treated with ATR inhibitor. WT wild-type. n = 3. Data are represented as mean +/− SEM. Students *t* test. ****p<0.0001, **p<0.0021, *p<0.032. **G** Schematic of tau’s nuclear mechanism.

In post-mitotic cells like neurons, radiation-induced injury triggers a cascade of cellular events, including oxidative stress, mitochondrial changes, and repair response that signal to other response proteins, especially for repair of damaged DNA [16, 40, 45]. Our IP-MS findings revealed unique interactions between tau and DDR proteins. To investigate tau’s role in DDR in the brain, we exposed wild-type and tau-knockout (TKO) mice to a 10 Gy whole brain radiation dose. Brain tissues collected 24 hours post-radiation showed decreased fluorescence intensity of gamma H2AX (γH2AX) DNA foci in TKO mice compared to controls (Fig. 3A–D). Moreover, pharmaceutical inhibition of Ataxia-telangiectasia-mutated (ATR), a crucial DDR regulator, abolished tau phosphorylation, suggesting ATR’s upstream regulation of tau (Fig. 3E–F). These results signify tau’s regulatory role in radiation-induced DDR response (Fig. 3G).

#### Translation machinery and protein synthesis is modulated in tau-knockdown hiPSC-derived cerebral organoids and tau-knockout mice after injury

**Figure 4:**
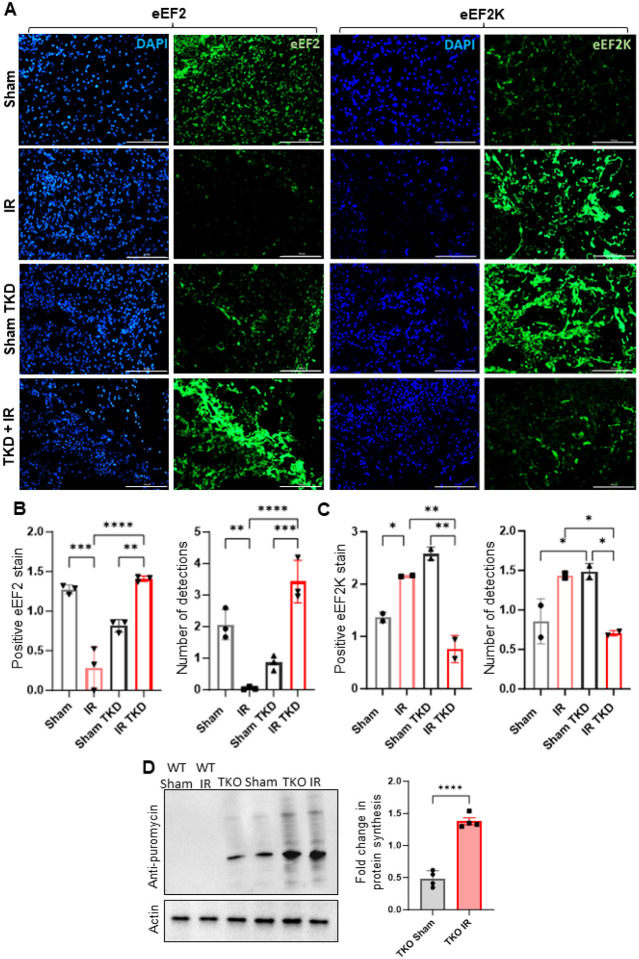
Translation machinery and protein synthesis is modulated in tau-knockdown hiPSC-derived cerebral organoids and tau-knockout mice after injury. **A**eEF2 in irradiated versus unirradiated (0 Gy) control. eEF2K levels changed in a manner opposite to eEF2. Scale bar:100 μm. Quantitative analysis of positive eEF2 (**B**) and eEF2K (**C**) staining and number of total proteins detected. n = 3 (eEF2) and n = 2 (eEF2K). Data are represented as mean +/− SEM. 1-way analysis of variance. ****p<0.0001, ***p<0.0002, **p<0.0021, *p<0.032. **D** SUnSET assay western blot analysis of protein synthesis in tau knockout (TKO) sham versus irradiated mice shows higher fold change in protein synthesis higher in irradiated TKO mice. IR, irradiation; TKD, tau knockdown. n = 3. Data are represented as mean +/− SEM. Students *t* test. ***p<0.0002.

Our IP-MS analysis indicated a loss of ptau interaction with eEF2 in comparison to unirradiated control. eEF2 is pivotal for protein synthesis regulating the elongation step in translation, a highly conserved process in both eukaryotes and prokaryotes [46]. Phosphorylation of eEF2 at Thr56, also known as eEF2K, is its inactivated form [47, 48]. Post-radiation, an increase in eEF2K was observed in hiPSC-derived cerebral organoids, signifying radiation-induced eEF2 inactivation through phosphorylation. Additionally, tau knockdown (TKD) led to elevated eEF2K levels in organoids, suggesting that loss of tau also inactivates eEF2. Strikingly, radiation-induced eEF2K upregulation was attenuated in the TKD condition, indicating TKD rescues eEF2 activation following radiation (Fig. 4A–C). This led to the hypothesis that rescuing eEF2 activation in TKD might enhance protein translation. Using the SUnSET assay [49] in TKO mice after radiation exposure, we observed increased protein translation at 24 hours compared to unirradiated controls (Fig. 4D), suggesting that loss of tau rescues eEF2 activation, impacting protein translation in response to neuronal injury.

### Synaptic composition is altered in response to injury and tau status

**Figure 5:**
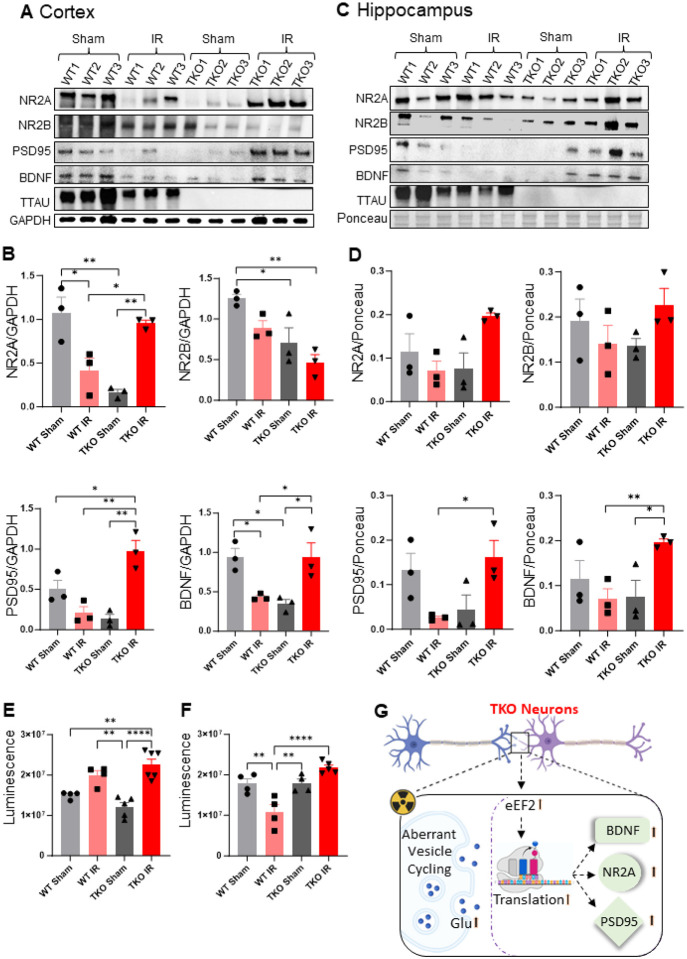
Synaptic composition is altered in response to injury and tau status. Western blotting images of pre- and post-synaptic proteins in synaptosomes isolated from mouse cortex (**A**) and hippocampus (**C**). Quantitative analysis of GRIN2A, GRIN2B, PSD95, and BDNF normalized to GAPDH (**B**) and Ponceau S stain (**D**). n = 3. Data are represented as mean +/− SEM. 1-way analysis of variance. **p< 0.0021, *p<0.032. (**E-F**) Glutamate levels recorded by luminescence across groups in both cortex (CX) and hippocampus (HP). n = 3. Data are represented as mean +/− SEM. 1-way analysis of variance. ****p<0.0001, **p<0.0021. **G** Schematic of tau’s synaptic mechanism.

Studies of neuronal dendrites and dendritic spines, the postsynaptic loci of most excitatory synapses in the brain, have shown evidence of eEF2-mediated translation of synaptic proteins in homeostatic conditions [50]. Given the role of eEF2 in irradiated neurons lacking tau, we hypothesized that radiation would lead to upregulation of both pre-synaptic and post-synaptic proteins (NMDAR subunits 2A and 2B (GRIN2A and GRIN2B)), brain-derived neurotrophic factor (BDNF), and post-synaptic density protein 95 (PSD95). To determine the role of tau in neuronal synaptic composition after irradiation, we exposed mice to a single 10 Gy dose of radiation and isolated synaptoneurosomes [51] from cortical and hippocampal samples for western blot analysis. We observed upregulation of GRIN2A, BDNF, and PSD95, but downregulation of GRIN2B, in the cortex at 24 hours after irradiation in TKO mice compared with sham and wild-type controls (Fig. 5A–B). BDNF and PSD95 levels also increased in the hippocampus after irradiation in TKO mice, but GRIN2A or GRIN2B levels did not change (Fig. 5C–D). Since effective synaptic plasticity and the strength of synaptic communications require a complex balance of pre- and post-synaptic proteins, these results highlight a previously unexplored role of tau in regulating synaptic homeostasis in injury.

### Neurotransmitter content and vesicle exocytosis/endocytosis at synapses are affected by radiation-induced injury and tau status

A key mechanism of synaptic homeostasis is the dynamic interplay between neurotransmitter flux and vesicle transport, critical for effective neurotransmission and maintaining physiological levels of glutamate at the neuronal synapse, which are in turn crucial functions of brain health [52]. Glutamate is the primary excitatory neurotransmitter in the mammalian brain [53]. To test glutamate levels, we isolated synaptoneurosomes from the cortex and hippocampus of irradiated and unirradiated mice and measured glutamate with a luminescence assay kit (Promega). In accordance with the pattern of synaptic protein changes resulting from irradiation and tau loss, glutamate levels increased in both the cortex and hippocampus of TKO mice exposed to radiation (Fig. 5E–F).

Glutamate and other neurotransmitters are packaged in vesicles and recycled at neuronal synapses [54]. High glutamate levels disrupt vesicular transport and perturb activity [54]. To measure the effects of irradiation and tau loss on vesicular exocytosis/endocytosis, we used FM 2–10 dye [55], which incorporates into docked vesicle membranes, undergoing fusions with rapid packaging of neurotransmitters before release into the synaptic gap [56]. Our results show that dye uptake and release was slowest in TKO mice exposed to radiation at up to 480 seconds after calcium activation in the cortex, hippocampus, and cerebellum compared with sham controls and wild-type control mice (Supplementary Fig. 4A-C). Collectively, these results indicate tau’s important role in maintaining synaptic homeostasis in neuronal injury (Fig. 5G).

### Radiation injury and tau knockout worsens cognitive functioning in mice

**Figure 6:**
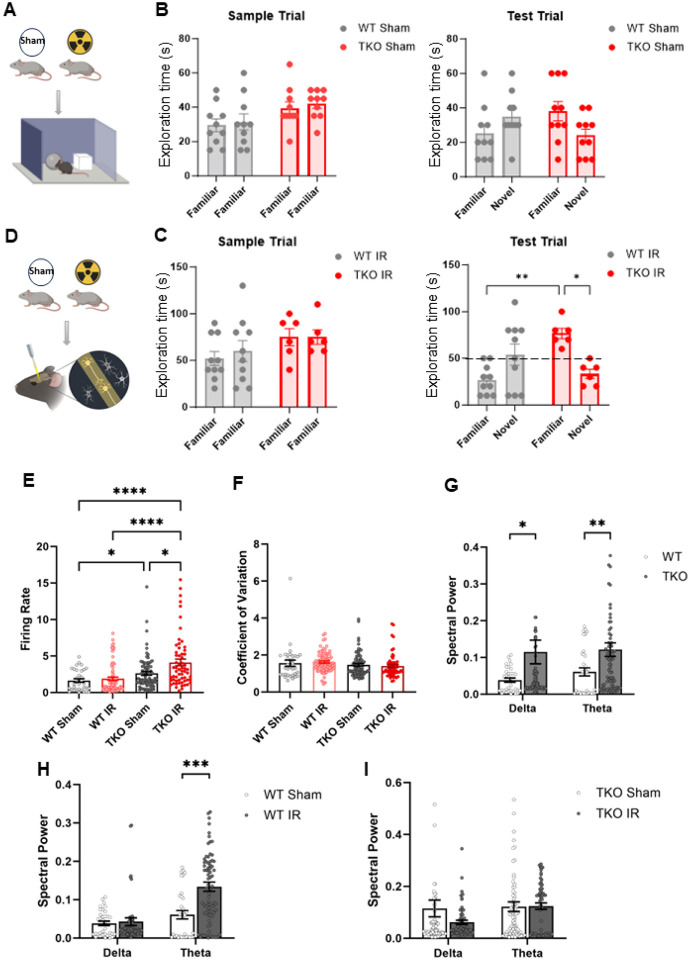
Radiation injury and tau knockout worsens cognitive functioning and impairs spontaneous firing patterns and spectral power in tau knockout mice. **A**Novel object recognition test in sample trial and test trial in tau knockout (TKO) versus wild-type (WT) mice. **B** Novel object recognition test in TKO and wild-type mice 5 days after irradiation. n = 7 TKO IR, n = 10 all other groups. Data are represented as mean +/− SEM. 2-way analysis of variance. **p< 0.0021, *p<0.032. **C**Firing rate is increased in irradiated TKO mice compared with WT. **D** No significant difference in coefficient of variation across groups. **E**Spectral power of EEG recordings to delta and theta waves are significantly increased in TKO mice compared with WT. **F** Spectral power is higher for theta waves in irradiated WT mice compared with sham, with no difference in delta waves. **G** Theta waves persist in irradiated TKO mice compared with sham, with no difference in delta waves. n = 4 each group. Data are represented as mean +/− SEM. 2-way analysis of variance. ***p<0.0002, **p<0.0021, *p<0.032.

After we observed synaptic changes resulting from radiation injury and loss of tau, we assessed cognitive profiles in TKO and wild-type mice (Fig. 6A). The novel object recognition test is widely used to assess recognition memory in rodents [57] and cognitively intact mice spend an increased amount of time with the novel object [57]. No significant difference in exploration time for the novel object was noted in either TKO or wild-type mice that had not been exposed to radiation (Fig. 6B). However, at 5 days after irradiation, the TKO mice showed significantly less exploration of the novel object than did irradiated wild-type mice (Fig. 6C), suggesting that tau participates in the neuroprotection of brain tissue injured by radiation.

### Radiation injury impairs spontaneous firing patterns and spectral power in tau knockout mice

The prefrontal cortex (PFC), through its extensive connections with other brain regions, regulates higher-order cognitive abilities [58]. We rst analyzed neuron firings within the PFC in wild-type and TKO mice using implanted electrodes (Fig. 6D) and found the firing rate in TKO mice to be markedly higher than that in the wild-type controls (Fig. 6E). Reasoning that the absence of tau may render PFC neurons more sensitive by introducing synaptic changes that would influence their firing activities, we tested the response of PFC neurons to radiation to further clarify the impact of tau on neuronal sensitization. Our previous experiments with wild-type rats demonstrated that irradiation led to transient increases in firing rate, with a return to baseline levels within weeks [17]. The current experiment revealed similar results in that no significant difference in firing rate was detected between unirradiated and irradiated wild-type mice 5-days following radiation exposure. In stark contrast, irradiated TKO mice showed prolonged elevation in the firing rate of PFC neurons (Fig. 6E) that persisted for up to 1 month. This finding underscores the heightened sensitivity of PFC neurons in tau-deficient mice to radiation. Despite the significant differences noted in firing rates in TKO mice, no discernible disparities emerged in the firing regularities of PFC neurons between TKO and wild-type mice, as revealed by the consistent coefficient of variation of the interspike intervals in all groups (Fig. 6F).

To obtain additional insight into the effects of tau deficiency on neural activity, we also evaluated local field potentials within the PFC by evaluating electroencephalogram (EEG) oscillations in the delta and theta bands, which are associated with cognitive and memory functions. Consistent with previous studies showing higher spectral powers in both delta and theta oscillations in patients with Alzheimer’s or Parkinson’s disease with dementia [59, 60], we observed increased oscillation powers in both the delta and theta EEG in TKO mice relative to wild-type mice (Fig. 6G), implying that the absence of tau may have cognitive effects. Notably, radiation exposure had distinct effects on spectral power within the theta band, in that wild-type mice showed increase in theta band spectral power after irradiation, indicating a neural response to radiation. Intriguingly, this effect was not replicated in TKO mice (Fig. 6H–I). This observation highlights the complexity of the interplay between tau and radiation exposure on neural activity dynamics.

## Discussion

We identified previously undefined subcellular-specific functions of tau in acute neuronal injury in multiple model systems that otherwise simulate normal physiological conditions. The effects of radiotherapy used for cancer treatment have generally focused on radiation-induced DNA damage, particularly double strand breaks [61]. Such damage activates a diverse array of repair machinery that prevents cell cycle progression at specific checkpoints thus allowing for DNA repair [62]. Some investigators have proposed that tau in the nucleus has a non-canonical neuroprotective function by maintaining and regulating heterochromatin, localizing with acrocentric chromosomes, binding with gene-coding and intergenic regions, and being active at pre-RNA processing sites [63]. Tau also participates in protecting neuronal DNA from damage through heat and oxidative stress [34]. In this study, we discovered a unique role for tau in radiation-induced neuronal DDR via direct interactions (evaluated via IP-MS) with key DDR proteins such as H2Ax.

In traumatic brain injury or neurodegenerative diseases, H2AX has been shown to be a sensitive marker of DNA damage through phosphorylation of its serine residue at Ser_139_ (γH2Ax) [64]. We found that γH2Ax foci were diminished in the absence of tau and in the presence of radiation-induced injury, and our ChIP-Seq findings show ptau enrichment in gene regions involved in DDR. Curiously, we detected basal levels of ptau in the nucleus before irradiation, which may reflect a homeostatic role of tau in normal neurons. In the event of an injury, ptau rapidly localizes and increases in the nucleus, setting in motion an injury response by interacting with proteins involved with repairing damaged DNA. In line with established functions of DDR markers in disease, our results suggest that tau acts as a regulator of the damage response in the injured brain.

In addition to its functions in the neuronal nucleus for damage repair, tau has critical functions in other neuronal compartments as well. Tau has a sirtuin-dependent role in protein synthesis [65]. In neurons, homeostatic translation requires the coordinated transportation of ribosomes, tRNAs, mRNAs, and other important machinery at proximal and distal sites of neuronal dendrites with pristine fidelity to avoid mistranslation and ribosomal frameshifting [66–72]. Even the subtlest changes in this finely tuned machinery can lead to misfunction [73] and conditions such as epileptic seizures and frontotemporal dementia [74–76]. We found that tau has a unique interaction with a crucial translation marker, eEF2. In cells with loss of tau and exposed to injury, active status of eEF2 is restored, indicating a shift in translation balance. This shift can have downstream repercussions, as shown in studies showing localized eEF2/eEF2K activity in dendrites, which affects glutamate signaling, downstream NMDAR activation, subsequent increase in calcium levels, and long-term depression [72, 74, 77–79]. Our results suggests that tau has a critical role in maintaining translation homeostasis via its interaction with eEF2. Indeed, regulation of eEF2 by tau could prevent superfluous protein synthesis and ensure translation of proteins essential for neuronal maintenance post-injury, especially because translation is an energy-demanding process. Moreover, the synthesis of synaptic proteins amplified in the absence of tau and injury, perhaps because of unchecked eEF2 active status. Taken together, these results suggest that tau has a regulatory role in the translation machinery of the injured brain.

The cumulative effects of radiation-induced insult have detrimental effects on neuronal and dendritic architecture [80–84]. Our lab has shown that cranial irradiation induces axon initial segment alternations and dysfunction in neurons [17]. Structural alterations such as these are detrimental for axonal transport and neuronal communication. We also observe reductions in tau levels (relative to the 0 Gy control) after irradiation in cortical (Supplementary Fig. 5A,D) and hippocampal (Supplementary Fig. 5B,E) neurons and hiPSC-derived cerebral organoids (Supplementary Fig. 5C,F). Others have shown that tau knockdown affects neuronal damage via growth cone impairment, delayed maturation, reduced microtubule density, and synaptic changes [85]. Our results show that radiation-induced injury results in decreases in pre- and post-synaptic proteins relative to controls. However, we also report that both loss of tau and neuronal injury led to increases in pre- and post-synaptic proteins GRIN2A, PSD95, and BDNF, and increases in glutamate and eEF2 levels. These findings lead us to hypothesize a complex “feed-forward” adaptive mechanism of higher synaptic translation, elevated glutamate content and hence increased post-synaptic protein expression in the absence of tau and the presence of neuronal injury, resulting in excitotoxicity and aberrant neuronal firing. Although the molecular basis of this relationship needs to be investigated further, our findings on cognitive performance and EEG spectral power in TKO mice exposed to neuronal injury suggest a role for tau as a neuroprotector. TKO mice performed worse in novel object recognition test after irradiation compared to wild-type control. Besides, persistent firing of neurons in the PFC during working memory tasks has been well documented to orchestrate cognitive dynamics [58]. In TKO mice, neuronal firing was aberrant for up to 1 month after injury, suggesting that tau has a sustained role in maintaining optimal firing in the injured brain long-term.

A limitation of our experimental approach is that our model relies on neuronal injury induced by a specific treatment modality used in clinic for cancer patients. Injury models often recapitulate a ‘pathology state’, which was not the aim here since our model lacked classical disease-specific properties observed in traumatic brain injury or stroke models. The strength of our study is using normal physiologically relevant systems across multiple species, namely rat cortical and hippocampal neurons, mouse whole brains, as well as human iPSCs to generate neurons in both 2D cultures and 3D cerebral organoid systems. All systems revealed the same tau response to radiation.

In this study, we present evidence that tau has an important function to play in vertebrate central nervous system in an acute response to ionizing radiation-induced injury. We provide evidence that tau directly interacts with proteins involved in brain homeostasis and participates in preventing cognitive decline. These findings provide a baseline to study the chronic aspects of tau in radiation-induced injury, as well as identify pathways for potential therapeutic targets.
